# Inhibition of Host Arginase Activity Against Staphylococcal Bloodstream Infection by Different Metabolites

**DOI:** 10.3389/fimmu.2020.01639

**Published:** 2020-07-28

**Authors:** Rui Pang, Hua Zhou, Yifeng Huang, Yubin Su, Xinhai Chen

**Affiliations:** ^1^Guangdong Provincial Key Laboratory of Microbial Safety and Health, State Key Laboratory of Applied Microbiology Southern China, Guangdong Institute of Microbiology, Guangdong Academy of Sciences, Guangzhou, China; ^2^Department of Respiratory and Critical Care Medicine, School of Medicine, The First Affiliated Hospital, Zhejiang University, Hangzhou, China; ^3^Key Laboratory of Functional Protein Research of Guangdong Higher Education Institutes, Department of Biotechnology, College of Life Science and Technology, Jinan University, Guangzhou, China; ^4^Shenzhen International Institute for Biomedical Research, Shenzhen, China; ^5^Department of Microbiology, University of Chicago, Chicago, IL, United States

**Keywords:** metabolite, *Staphylococcus aureus*, bloodstream infection, arginase, nitric oxide

## Abstract

*Staphylococcus aureus* is a notorious bacterial pathogen that often causes soft tissue and bloodstream infections and invariably garners resistance mechanisms against new antibiotics. Modulation of the host immune response by metabolites is a powerful tool against bacterial infections, but has not yet been used against *S. aureus* infections. In this study, we identified four metabolite biomarkers: L-proline, L-isoleucine, L-leucine, and L-valine (PILV), through a metabolomics study using animal models of *S. aureus* bloodstream infection. The exogenous administration of each metabolite or of PILV showed anti-infective effects, and a higher protection was achieved with PILV in comparison to individual metabolites. During the staphylococcal infection, the expression of most host arginase and nitric oxide synthase (NOS) isozymes was simultaneously induced in mouse liver, kidney, and blood samples. However, the induction of arginase isozymes was dramatically stronger than that of NOS isozymes. This elevated arginase activity was inhibited by the metabolite biomarkers thus killing *S. aureus*, and PILV exhibited the strongest inhibition of arginase activity and bacterial inhibition. The suppression of arginase activity also contributed to the metabolite-mediated phagocytic killing of *S. aureus* in mouse and human blood. Our findings demonstrate the metabolite-mediated arginase inhibition as a therapeutic intervention for *S. aureus* infection.

## Introduction

*Staphylococcus aureus* is both, a human commensal and a significant cause of hospital- and community-acquired diseases, including soft tissue infections, pneumonia, osteomyelitis, septic arthritis, bacteremia, endocarditis, and sepsis (1–3). Among the invasive strains isolated from the blood of patients with *S. aureus* bacteremia, 80% are genetically indistinguishable from the nasal strains detected at the moment of admission (4). Due to the high incidence of hospital-acquired infections, antibiotics are used for *S. aureus* decolonization and for prophylaxis of nosocomial disease (5, 6). However, the emergence and spread of drug-resistant strains, notably methicillin-resistant *S. aureus* (MRSA), lead to increased therapeutic failure and mortality rates (6), and therefore, new approaches are needed for treating such infections in the clinic. One possible strategy would be to enhance the innate immune response of the infected host, thereby restoring its ability to kill the bacterial pathogen in a relatively risk-free manner (7).

*S. aureus* infection causes several expressive metabolism changes in the host, including oxidative phosphorylation, aerobic glycolysis, and amino acid and fatty acid metabolisms (8–10). These altered pathways have two leading roles: to facilitate bacterial invasion or to benefit the immune response against the infection. The host central carbon metabolism is strongly disturbed by *S. aureus*, which activates autophagy by increasing phosphorylation of AMP-activated protein kinases and extracellular signal-regulated kinases, thereby meeting the staphylococcal invasion (11). Furthermore, the internalization of *S. aureus* destroys the host's arginine metabolism and limits the production of nitric oxide, which serves in the host antibacterial defense, eventually inducing cell death (12, 13). In contrast, studies focusing on the cross-talk between metabolic regulation and the immune system reveal an active role of metabolic regulation in the control of pathogenic bacteria. In several bacterial infection models, hosts that survive the infection display distinctive metabolic pathways (14–18). Many of the metabolites of these metabolic pathways are immunoregulators that act via various mechanisms including the activation of PI3K/Akt1, elevated expression of cytokines, and NO production (14–18). To the best of our knowledge, the modulation of host innate immunity by metabolites has not been demonstrated as a valuable strategy against staphylococcal infection thus far.

Here, we used a gas chromatography-mass spectrometry (GC-MS) technique to identify metabolites from BALB/c mice infected by three sublethal doses of *S. aureus* strain Newman. The results suggest that four metabolites (L-proline, L-isoleucine, L-leucine, and L-valine) target NO production in order to kill *S. aureus* and may aid in the development of therapeutic interventions that can improve the outcome of staphylococcal infections.

## Materials and Methods

### Bacterial Strains, Culture Conditions, and Experimental Animals

*S. aureus* strains Newman (ATCC 25904), USA300 (ATCC BAA-1717), or MRSA252 (ATCC BAA-1720) were cultured in tryptic soy agar (TSA) at 37°C. A single colony was grown in tryptic soy broth (TSB) at 37°C overnight. The cells were washed and re-suspended in sterile phosphate-buffered saline (PBS). Female BALB/c mice (6 weeks old) were reared in specific pathogen-free (SPF) facilities and fed with dry pellet diets and sterile water. The mice were kept at 20–24°C, 45–65% relative humidity and a light/dark cycle of 12/12 h in group housings. Each mouse was then intravenously infected with a low (0.3 × 10^7^), moderate (0.7 × 10^7^), or high (1 × 10^7^) concentration (colony-forming units, CFU) of *S. aureus* Newman or with sterile PBS (no infection group) according to previous studies (19, 20). Samples of 50 μL blood were collected from the orbital vein of each mouse 12 h post-infection. Animal research was approved by the Institutional Animal Care and Use Committee at the Guangdong Institute of Microbiology (Animal Welfare Assurance Number GT-IACUC201907031).

### Metabolomics Analysis

The metabolite extraction was performed following methods described previously (18). Briefly, 50 μL plasma was quenched using 50 μL cold methanol and the supernatant was collected by centrifugation at 8,000 rpm for 3 min. Subsequently, Ribitol (5 μL, 0.1 mg/mL) was added as an internal analytical standard, and samples were dried. Sample derivatization and the subsequent data analysis were carried out as described previously (17, 18). Samples were oximated and derived, and then analyzed by GC-MS (Trace DSQ II, Thermo Scientific, Waltham, USA). Two technical replicates were conducted for each sample. The metabolomics data was deposited to FigShare (https://doi.org/10.6084/m9.figshare.12366554).

For data processing, spectral deconvolution and calibration were performed using the automated mass spectral deconvolution & identification system (AMDIS) and internal standards. Metabolites from the GC-MS spectra were identified by the National Institute of Standards and Technology (NIST) MS search 2.0. The resulting normalized peak intensities formed a single matrix with Rt-m/z pairs for each file in the dataset. The data were median centered and inter-quartile ranges (IQR) were scaled per sample (21). ClustVis was employed to create a principal component analysis (PCA) plot and heatmaps (22). Metabolic pathways were enriched using MetaboAnalyst 4.0 (23).

### Effect of Metabolites on *S. aureus* Infection

BALB/c mice were randomly divided into groups for investigating the effect of L-proline, L-isoleucine, L-valine, L-leucine, or a mixture of all four metabolites (designated as PILV). Hundred microliter of a solution containing each metabolite (0.5 g kg^−1^) or an equal volume of sterile saline solution (no metabolite control) were intraperitoneally injected into each mouse. After 6 h, mice were intravenously challenged by *S. aureus* Newman (1 × 10^7^ CFU/mouse) and continued receive the metabolites once a day. Body weight was measured daily. All mice were euthanized 15 days post-infection and the kidneys were separated. The bacterial load in each organ was detected by homogenizing, diluting and sampling on TSA. The remaining organ was investigated by histopathology analysis (24). For survival, BLAB/c mice were intravenously inoculated with 100 μl of bacterial suspension at a concentration of 2 × 10^8^ CFU ml^−1^ (USA300) or 2 × 10^9^ CFU ml^−1^ (MRSA252). Each metabolite, PILV, BEC [S-(2-boronoethyl)-L-cysteine, an arginase inhibitor at 50 mg kg^−1^], or both a NO inhibitor (L-NMMA or L-NAME, 40 mg kg^−1^) and PILV were administrated as mentioned. PILV was administrated with 100 μl PBS at a concentration of 0.5 g kg^−1^ of each metabolite. Survival was monitored over 14 days.

### Determination of Arginase Activity, NO Release, and Urea

NO release in serum or tissues was calculated by examining the nitrate and nitrite concentrations with a Total Nitric Oxide Assay kit (Beyotime, Haimen, China). Optical densities at 540 nm were verified using a microplate reader (Biotek Instruments, Winooski, USA) and NO concentrations were calculated from a standard curve. Urea production in serum was determined using a Urea Colorimetric Assay kit (BioVision, Milpitas, USA). Mouse serum, kidney and liver were collected for the Arginase Activity Assay kit (MAK112, Sigma-Aldrich, San Luis, USA) according to the manufacturer's instructions.

### Quantitative Real Time-Polymerase Chain Reaction (qRT-PCR)

Total RNA was isolated from blood and tissues using TRIzol reagent (Invitrogen, Carlsbad, USA). The cDNA was synthesized using the PrimeScript^TM^ RT reagent Kit with the genomic DNA Eraser (Takara, Kusatsu, Japan). The mRNA levels of genes *Arg1, Arg2, Nos1, Nos2*, and *Nos3* were detected using qRT-PCR with TB Green™ Premix Ex Taq™ II (Takara) in a LightCycler96 system (Roche, Penzberg, Germany). The housekeeping gene coding for β*-*Actin (*Actb*) was used as an endogenous control. All primers are listed in Table S1. After three repeated PCR reactions, the gene expression levels were calculated using the 2^−ΔΔCT^ method (25).

### Bacterial Survival in Human and Mouse Blood

All experiments using blood from human volunteers were approved by the Guangdong Institute of Microbiology's Institutional Review Board (IRB), and informed consent was obtained from all participants. Fresh human blood was collected with heparin. A volume of 0.45 ml of blood was pretreated with 50 μl of heat-killed *S. aureus* Newman (5 × 10^5^ CFU) at 37°C for 30 min and then mixed with 50 μl of a live 5 × 10^6^-CFU bacterial suspension in the presence or absence of PILV (10 mM for each metabolite), NO inhibitor (L-NMMA or L-NAME), or both. For mouse blood studies, 100 μl of heat-killed *S. aureus* Newman (5 × 10^5^ CFU) were intravenously injected into BALB/c mice and blood was collected by cardiac puncture after 6 h. A live bacterial suspension (50 μl) including 5 × 10^5^ CFU *S. aureus* Newman was mixed with 0.45 ml of mouse blood in the presence or absence of PILV (10 mM for each metabolite), NO inhibitor (L-NMMA or L-NAME), or both. All samples were incubated at 37°C with slow rotation for 60 min. Each sample then received 0.55 ml of lysis buffer (0.5% saponin, 200 U streptokinase, 100 μg trypsin, 2 μg DNase, 10 μg RNase per ml PBS) and was incubated for 10 min at 37°C before plating on TSA (19, 20).

### Cell Culture and Quantitative Phagocytosis Assay

The murine macrophage cell line RAW264.7 was cultured in Dulbecco's Modified Eagle Medium (DMEM) supplemented with 10% (V/V) fetal bovine serum (FBS, HyClone, Logan, USA). The human macrophage cell line U937 was grown in Roswell Park Memorial Institute (RPMI) 1640 medium supplemented with 10% FBS. All cells were grown in a 37°C incubator with 5% CO_2_. U937-derived macrophages were induced by 160 nM phorbol 12-myristate 13-acetate (PMA) at 37°C for 48 h. Macrophage phagocytosis was investigated as described previously (17, 18). After pretreatment with PILV, NO inhibitor, or both for 6 h, fluorescein (FITC)-conjugated *S. aureus* was centrifuged onto RAW264.7 or U937-derived macrophages at a multiplicity of infection (MOI) of 100. After 1 h of incubation, the macrophages were washed at least four times in cold PBS and then fixed in 4% paraformaldehyde before harvesting with cold PBS containing 5 mM ethylenediamine tetraacetic acid (EDTA). Finally, cells were subjected to fluorescence-activated cell sorting (FACS) analysis.

### Statistical Analysis

The relative abundances of metabolites in different groups were analyzed with one-way ANOVA (analysis of variance). Staphylococcal survival in blood, NO and urea levels, and macrophage phagocytosis were analyzed with the two-tailed Student's *t*-test. Bacterial loads and abscess numbers in renal tissues were analyzed with the two-tailed Mann–Whitney test. Statistical significance of survival curves was evaluated by long-rank test. All data analysis was performed with GraphPad Prism 8 (GraphPad Software, Inc.), and *P* < 0.05 were considered statistically significant.

## Results

### GC-MS-Based Metabolomics Identifies Host Metabolites Related to *S. aureus* Infection

**Figure 1:**
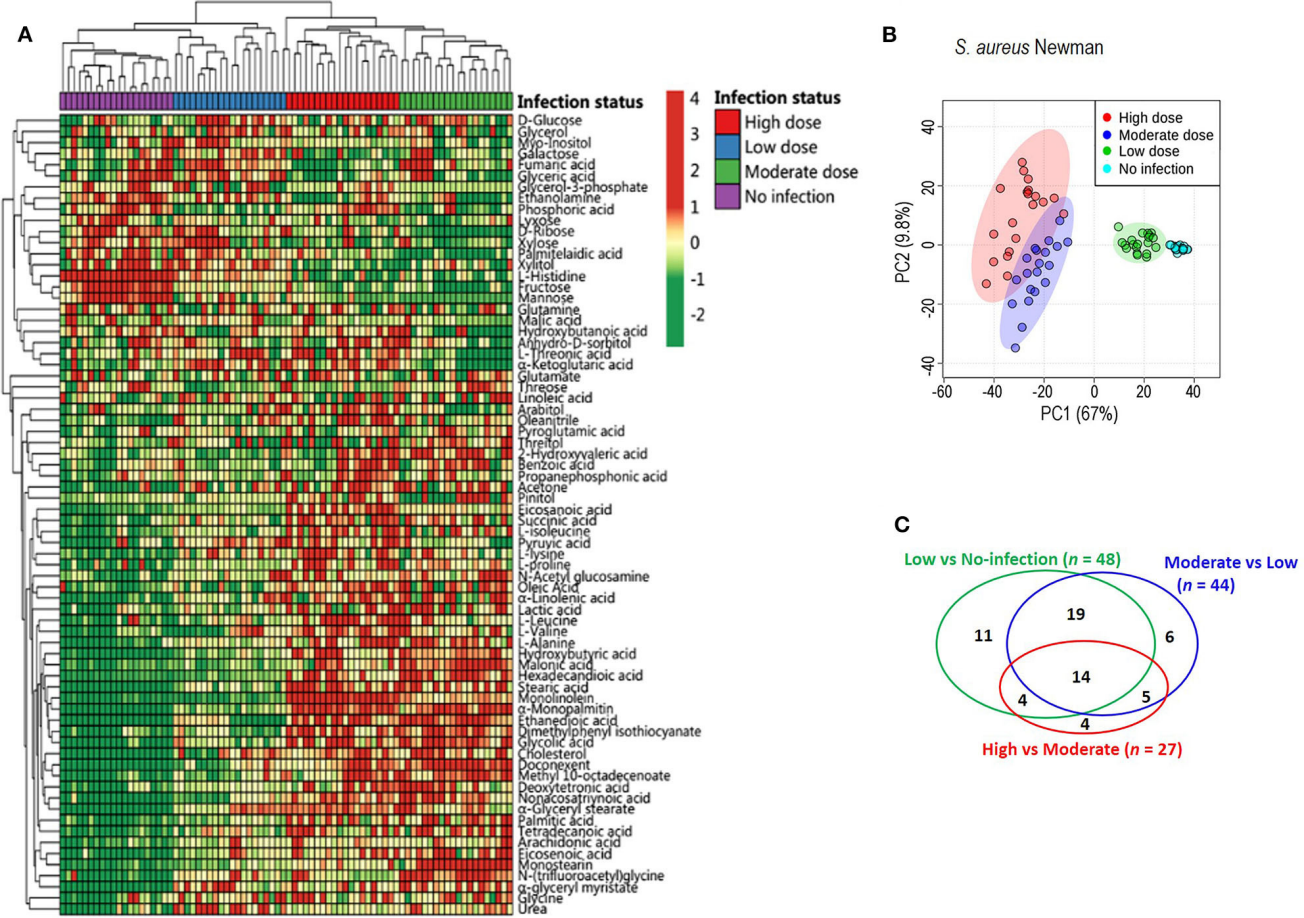
Metabolic profiles of mouse sera injected with different doses of *S. aureus* Newman. **(A)** The heat map for relative abundances of differential metabolites in low, moderate, and high dose of *S. aureus* Newman strain compared with no-infection control (*n* = 10, technical replicate = 2). Red and green indicated the increased and decreased of metabolite, respectively (see color scale). **(B)** Principal component analysis (PCA) of metabolomics data. Each dot showed a technical replicate for one sample. **(C)** Venn diagram showing the number of significant metabolites between pairwise comparison from no-infection group to high-dose group.

**Figure 2:**
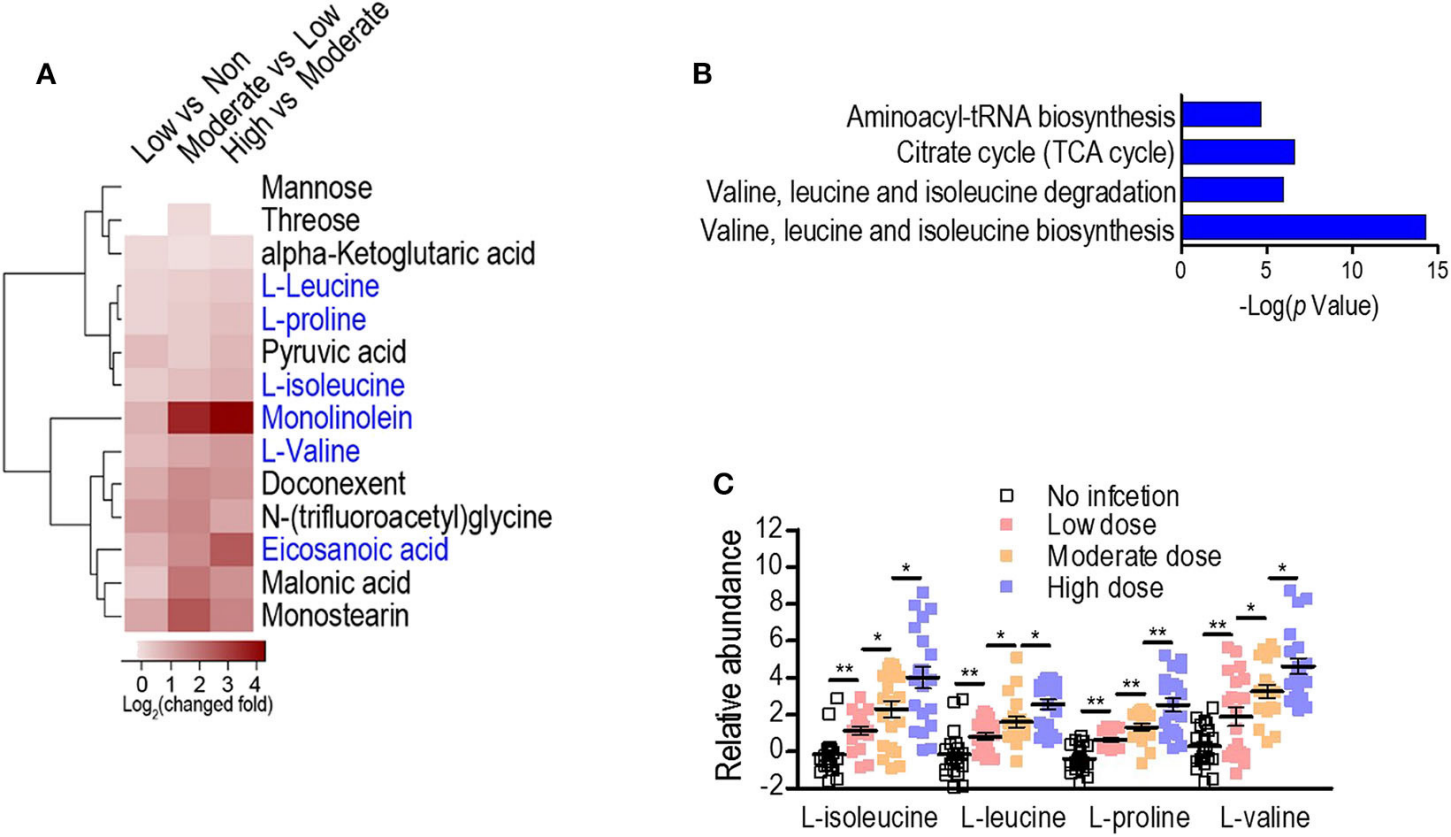
Serum metabolome analysis reveals L-isoleucine, L-leucine, L-proline, and L-valine as potential anti-infection metabolites against *S. aureus* infection. **(A)** Heat map representation of unsupervised hierarchical clustering of significant metabolites. **(B)** Pathway enrichment analysis of the differential metabolites. A horizontal histogram shows the impact of the enriched pathway with *P* < 0.01. **(C)** The abundance of L-isoleucine, L-leucine, L-proline, and L-valine in no infection, Low, Moderate, and High dose groups. Error bars ± SEM (*n* = 10, technical replicate = 2), *P < 0.05 and **P < 0.01 (one-way ANOVA).

To exploit anti-infection metabolites, metabolic profiling with different degrees of anti-infection was performed. We hypothesized that different sublethal infection doses would induce different degrees of anti-infection response by the host. Therefore, each group of BALB/c mice (*n* = 10) was intravenously challenged with low, moderate, or high doses of *S. aureus* Newman or with PBS, and a GC-MS-based approach was used to identify plasma metabolites in challenged mice. All mice survived without signs of toxicity or adverse events. A total of 72 metabolites were detected and displayed as a heat map (Figure 1A). The majority of metabolites suffered abundant changes. Results of PCA were able to distinguish no infection, low dose, moderate dose, and high dose groups (Figure 1B), demonstrating that hosts infected by different sublethal doses displayed differential anti-infection metabolic profiles. When comparing no-infection and low-dose groups, low-dose and moderate-dose groups, and moderate-dose and high-dose groups, 48, 44, and 27 differential metabolites were detected, respectively (Figure 1C). Among these, 14 differential metabolites were common to all comparisons. A subset of six metabolites (L-leucine, L-proline, L-isoleucine, monolinolein, L-valine, and eicosanoic acid) was significantly increased along with the infection dose (Figure 2A). In addition, the 14 differential metabolites shared among all dose comparisons enriched for four pathways: aminoacyl-tRNA biosynthesis; citrate cycle; valine, leucine, and isoleucine degradation; and valine, leucine, and isoleucine biosynthesis (*P* < 0.05) (Figure 2B). Out of the six metabolite biomarkers, L-leucine, L-proline, L-isoleucine, and L-valine were enriched in the following pathways: aminoacyl-tRNA biosynthesis; valine, leucine, and isoleucine degradation; and valine, leucine, and isoleucine biosynthesis. Therefore, L-leucine, L-proline, L-isoleucine, and L-valine were chosen for further investigation (Figure 2C).

### Exogenous Metabolites Display Anti-infective Effect on *S. aureus* Infection

**Figure 3:**
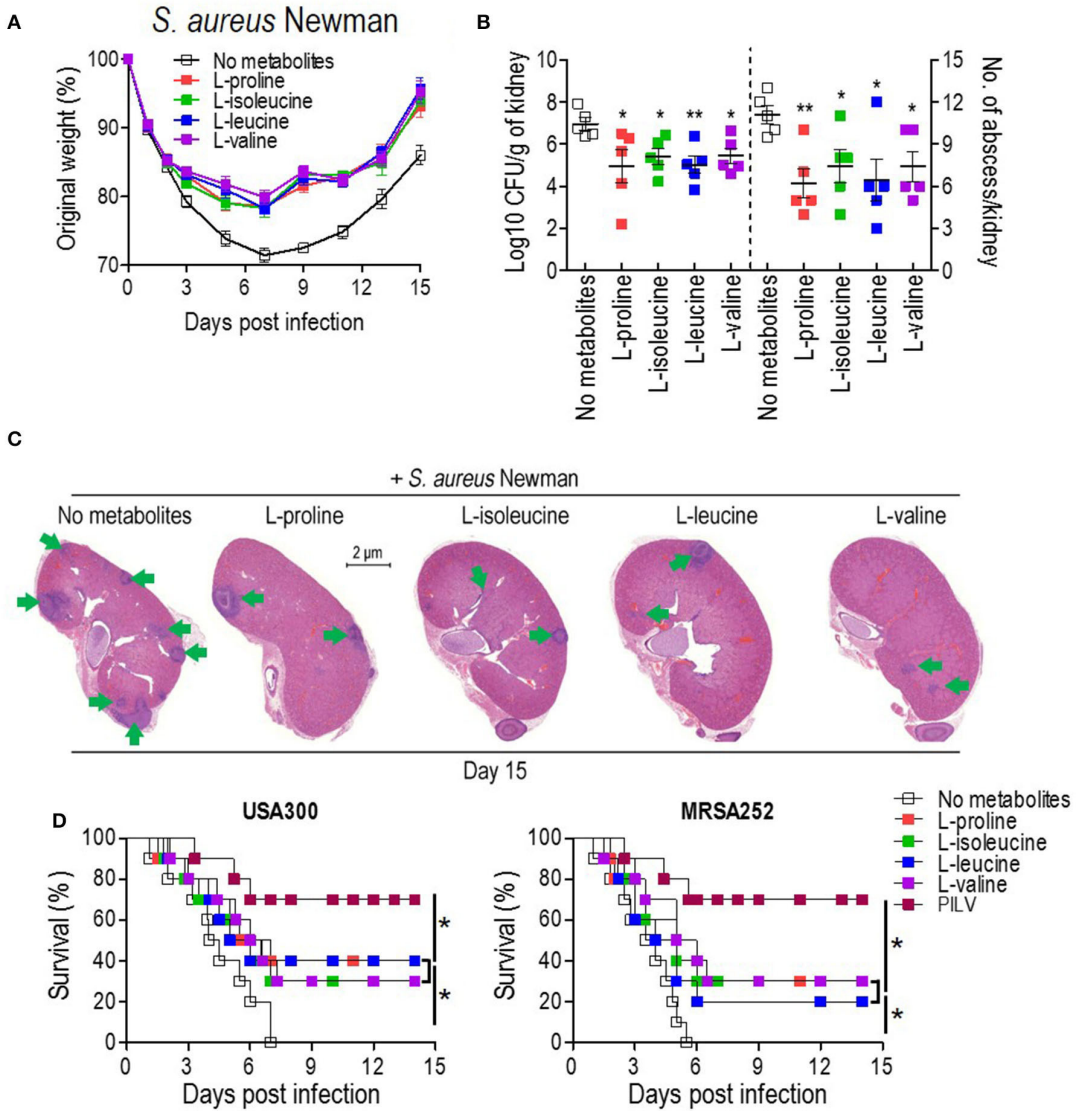
The administration of single metabolite or of a metabolite combination (named PILV) protects mice against *S. aureus* bloodstream infection. **(A–C)** Treatment with each metabolite, L-proline, L-isoleucine, L-leucine, or L-valine, rescued body weight loss **(A)** and reduced renal bacterial loads and abscess numbers **(B,C)** in mice infected by *S. aureus* Newman strain. Weight was recorded daily and reported as % of initial weight. Fifteen days post-infection, kidneys (*n* = 5) were removed and either ground for enumerating CFU/g tissue or fixed for counting surface abscesses **(B)**. Fixed kidneys were additionally thin sectioned and then stained with hematoxylin and eosin (H&E) for internal abscesses **(C)**. Green arrows point to internal abscesses in the kidney. **(D)** Treatment with single metabolites or a metabolite combination (PILV) protected mice (BALB/c, *n* = 20) against lethal bloodstream infection with *S. aureus* USA300 and MRSA252. Survival was monitored over 14 days. Data are represented as means ± SEM. *P < 0.05 and **P < 0.01 (two-tailed Mann–Whitney test).

To examine the anti-infective role of L-leucine, L-proline, L-isoleucine, or L-valine *in vivo*, cohorts of mice were intravenously infected with *S. aureus* Newman (1 × 10^7^ CFU) and injected with each metabolite (0.5 g kg^−1^) or sterile saline (no metabolite control) daily. When compared to the control group, metabolite-injected mice had significantly reduced weight loss, and staphylococcal load and abscess lesion in kidney tissues (two key disease outcome measures) during the *S. aureus* infection (Figures 3A–C). Each metabolite administration also provided distinct protection against a lethal bloodstream infection with USA300 (2 × 10^8^ CFU) and MRSA252 (2 × 10^9^ CFU) (Figure 3D). There was no difference in body weight recovery, bacterial loads, abscess numbers, and survival among all metabolites (Figures 3A–D). More importantly, the combined administration of all metabolites (PILV) was capable of promoting longer survival (Figure 3D), which was not reached by the administration of a 4-fold concentration of L-proline (2.0 g kg^−1^) (Figure S1); this indicated a synergistic effect of the four metabolites against the staphylococcal infection. Notably, the PILV combination treatment had no influence on the body weight and kidney morphology of mice without *S. aureus* infection (Figure S2). These data suggest that L-leucine, L-proline, L-isoleucine, or L-valine have anti-infective functions during *S. aureus* infection, and a PILV combination treatment could further improve the outcome of a MRSA infection.

### Metabolites Inhibit Arginase Activity to Enhance NO Production *in vivo*

**Figure 4:**
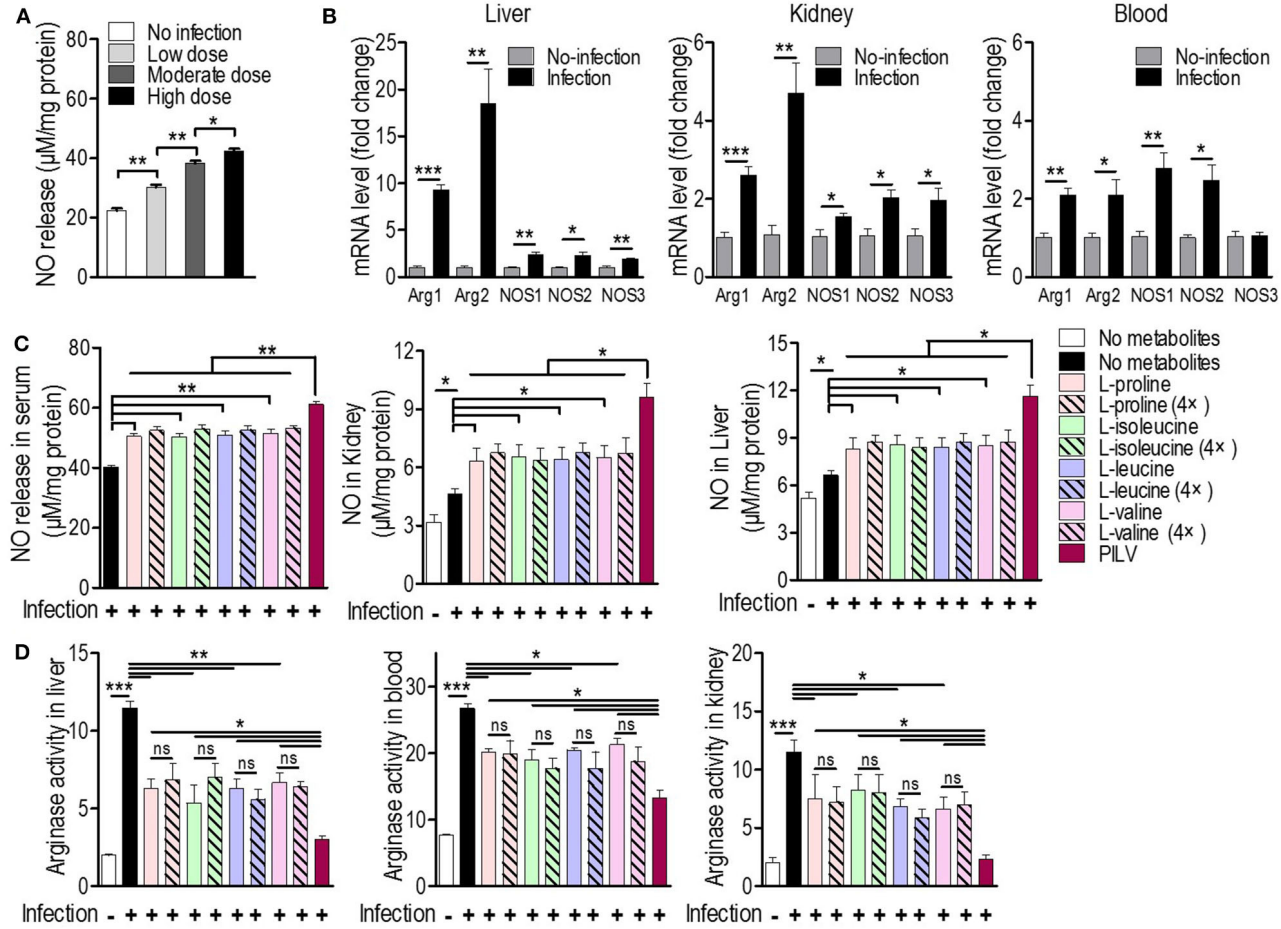
Metabolites promote NO production by arginase inhibition in mice. **(A)** The NO release under three sublethal doses (0.3 × 10^7^, 0.7 × 10^7^, and 1 × 10^7^ CFU/mouse) of *S. aureus* Newman infection (*n* = 5 for each dose group). Three days post-infection, mouse serum was collected and subjected to measurement of NO release. **(B)** The mRNA levels of two arginase (ARG) and three NO synthase (NOS) isoforms in mouse liver, kidney, and blood under a sublethal staphylococcal infection. *S. aureus* USA300 (5 × 10^6^ CFU/mouse) was used to infect BALB/c mice (*n* = 5). Three days after the infection, the mouse liver, kidney, and blood were collected and the transcriptional expression of ARG and NOS was measured. *Arg1* and *Arg2* are cytoplasmic and mitochondrial arginases, respectively. *Nos1, Nos2*, and *Nos3* are neuronal, inducible, and endothelial NOS, respectively. The NO production **(C)** and arginase activity **(D)** in mouse blood and tissues treated with single metabolite or PILV under staphylococcal infection. Data are represented as means ± SEM. *P < 0.05, **P < 0.01 and ***P < 0.001 (two-tailed Student's *t*-test).

Since L-valine can increase NO production in macrophages by inhibiting arginase activity (18), we asked whether L-leucine, L-proline, L-isoleucine, L-valine, or PILV could enhance NO production by blocking the arginase activity in *S. aureus*-challenged mice (*n* = 5 in each group). Firstly, we investigated whether a staphylococcal infection induced both ARG and NOS expressions simultaneously. NO production in serum samples and the expression levels of two ARG (cytoplasmic and mitochondrial arginases, designated as *Arg1* and *Arg2*) and three NOS isozymes (neuronal, inducible, and endothelial NOS, designated as *Nos1, Nos2*, and *Nos3*) were measured in tissues and blood upon *S. aureus* infection. Three days after a sublethal infection with *S. aureus* Newman, NO production was enhanced in a dose-dependent manner (Figure 4A). Furthermore, the intravenous infection with *S. aureus* USA300 triggered the expression of all arginase and NOS isozymes and increased NO production and arginase activity in mouse tissues (liver and kidney) and blood (or serum). *Nos3* expression was exceptionally unchanged in blood (Figures 4B–D). More interestingly, the *S. aureus* infection induced the expression of ARG isozymes in tissues more strongly than NOS isozymes (Figure 4B). This suggests that both ARG isozymes are predominant regulators of L-arginine, since ARG and NOS compete for L-arginine as an enzyme substrate (26). Cohorts of mice received a daily intraperitoneal injection of each metabolite (0.5 or 2.0 g kg^−1^) or PILV (0.5 g kg^−1^ of each metabolite) and were infected by *S. aureus* 6 h after the first injection. On day 3, animals were euthanized, and their blood and tissues (kidney and liver) were collected for measurements of NO production, arginase activity, and urea levels. The mice that received PILV showed the highest levels of NO in sera and tissues and the lowest arginase activities and urea levels in sera, followed by those that received one metabolite or a 4-fold concentration of that metabolite (Figures 4C,D). In the absence of a *S. aureus* infection, 0.5 or 2.0 g kg^−1^ of each metabolite or PILV (0.5 g kg^−1^ of each metabolite) showed no impact on NO production (Figure S3). During the staphylococcal infection, urea content was reduced by single-metabolite treatment and further decreased by PILV treatment (Figure S4A). These data suggest that L-leucine, L-proline, L-isoleucine, or L-valine can strengthen NO production, and PILV combination therapy has an additive effect on NO production through a stronger inhibition of arginase.

### Metabolite-Mediated Arginase Inhibition Protects Mice Against *S. aureus* Infection

**Figure 5:**
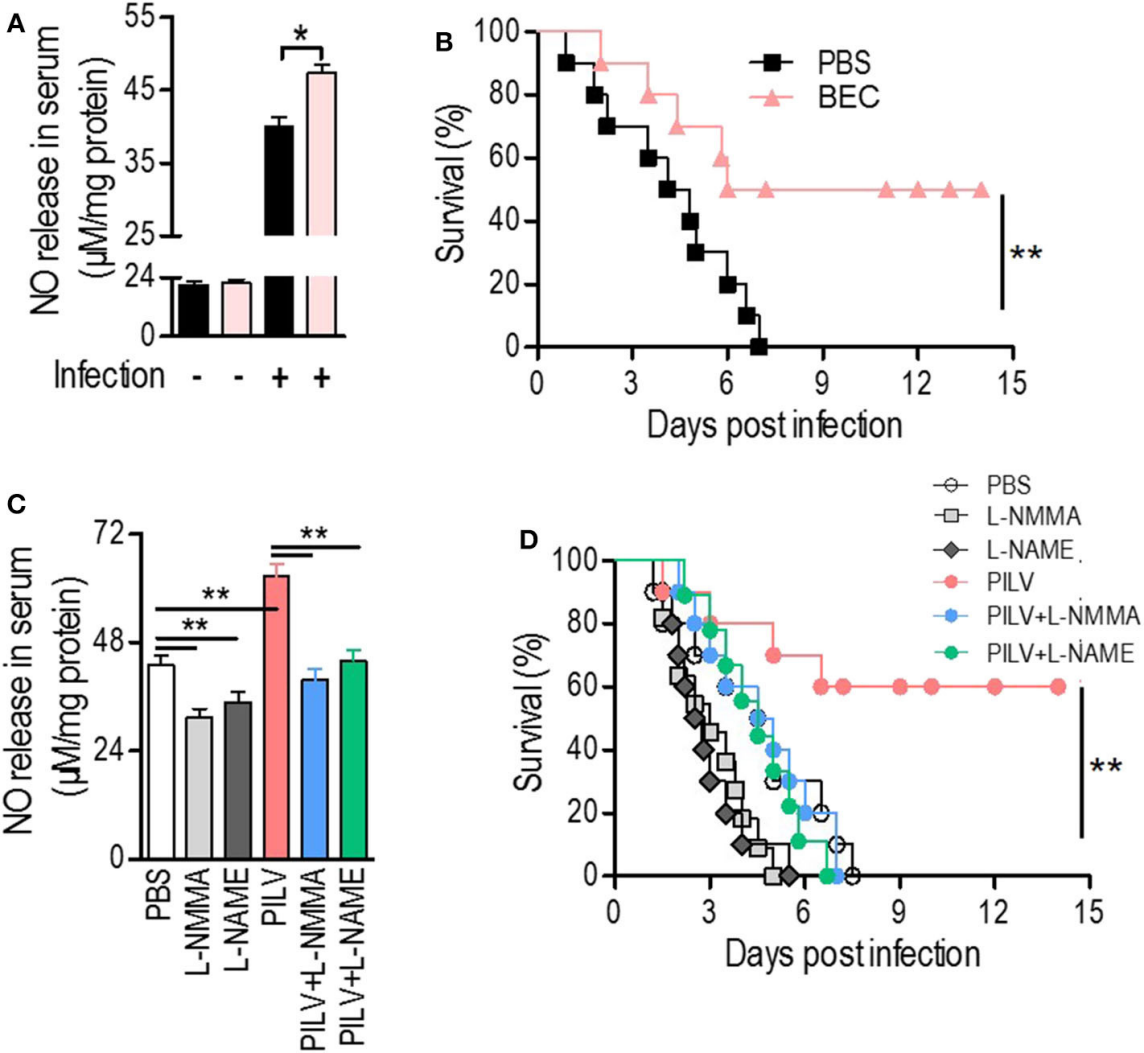
Metabolite-induced NO confers protection against staphylococcal infection. **(A,B)** The impact of a competitive arginase inhibitor, BEC (S-(2-boronoethyl)-L-cysteine), on NO release in serum **(A)** and survival **(B)** of mice under a lethal bloodstream infection with *S. aureus* USA300. **(C,D)** The impact of NO inhibitors, L-NMMA, and L-NAME, on NO release in serum **(C)** and survival **(D)** of mice under a lethal bloodstream infection with *S. aureus* USA300. Six hours after the injection of BEC (50 mg kg^−1^), L-NMMA (40 mg kg^−1^), L-NAME (40 mg kg^−1^), PILV, PILV plus L-NMMA (40 mg kg^−1^), or PILV plus L-NAME (40 mg kg^−1^), BALB/c mice (*n* = 30) were lethally challenged by *S. aureus* USA300 and then divided into two subgroups. One subgroup (*n* = 10) was used for NO measurement and another (*n* = 20) was used for observing survival. Five of the survival mice were euthanized 3 days after the infection to measure NO and urea production in serum. Survival was monitored over 14 days. Data are represented as means ± SEM. *P < 0.05 and **P < 0.01 (two-tailed Student's *t*-test).

Considering the positive correlation between infective protection and arginase inhibition after PILV treatment, we hypothesized that PILV-induced arginase inhibition could be responsible for a higher protection. We therefore tested this effect using a competitive arginase inhibitor, BEC, which showed no effect on NO production and urea levels under physiological conditions but enhanced NO production (Figure 5A) and decreased urea levels in serum samples of *S. aureus*-infected mice (*n* = 10 for each group) (Figure S4B). The survival assay indicated that BEC protected against lethal challenge with MRSA strain USA300 (Figure 5B). These data show that increasing NO production was beneficial in case of a MRSA infection. We then investigated the effects of two NO inhibitors, l-NMMA, and l-NAME, on PILV-induced NO production, and survival. As expected, the inhibitors significantly suppressed NO production induced by *S. aureus* infection in the absence or presence of PILV (Figure 5C). The mouse survival caused by *S. aureus* infection and enhanced by PILV was reduced by the NO inhibitors (Figure 5D). Together, these data indicate that PILV-induced arginase inhibition and subsequent NO production are able to confer protection against staphylococcal diseases.

### Metabolites Increase Phagocytic Killing of *S. aureus* in a NO-Dependent Manner via Arginase Inhibition

**Figure 6:**
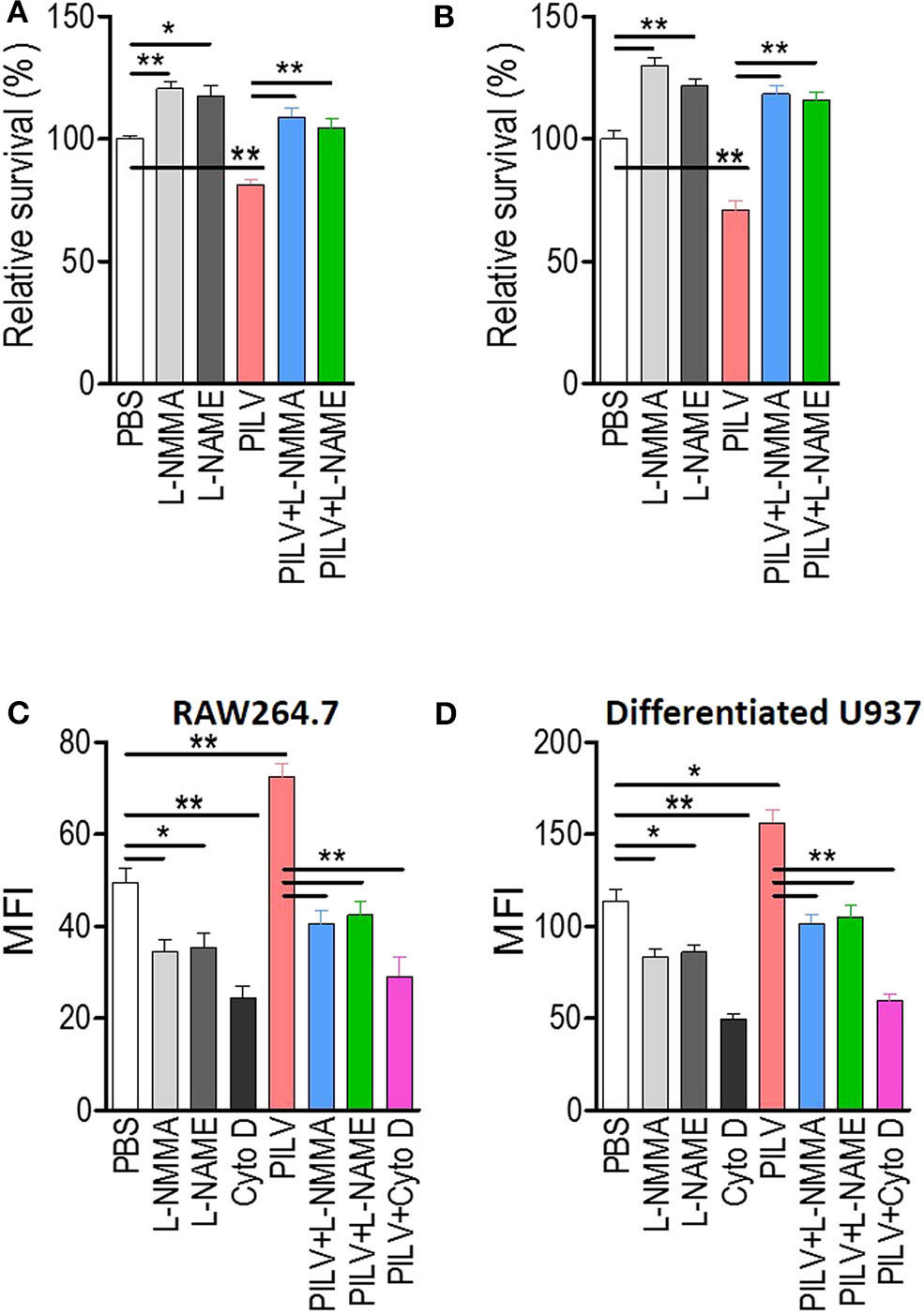
PILV enhances opsonophagocytic killing (OPK) in a NO-dependent manner. **(A,B)** Relative survival about OPK of *S. aureus* in human **(A)** and mouse blood **(B)** without or with PILV. Human and mouse anticoagulated blood treated with heat-killed *S. aureus* Newman was incubated with live *S. aureus* Newman (2.5 × 10^6^ CFU/ml blood for the human assay, 2.5 × 10^5^ CFU/ml blood for the mouse assay) in the presence of PBS, NO inhibitors, PILV, or PILV plus NO inhibitors for 60 min, and survival was measured (*n* = 5). **(C,D)** Determination the phagocytosis of FITC-conjugated *S. aureus* Newman in RAW 264.7 **(C)** and U937-derived macrophages **(D)** without or with PILV. RAW264.7 or U937-derived macrophages were pretreated with PBS, NO inhibitors, PILV, or PILV plus NO inhibitors in a serum-starved medium for 6 h and then were co-incubated with FITC-conjugated *S. aureus* for one more hour. Bacterial uptake was measured by flow cytometry. Data are represented as means ± SEM. *P < 0.05 and **P < 0.01 (two-tailed Student's *t*-test).

Next, we investigated if PILV had a function in human blood. *S. aureus* opsonophagocytic killing (OPK) was measured in human blood infected with 5 × 10^6^ CFU Newman for 60 min. Before the measurement, blood was pretreated with heat-killed *S. aureus* Newman for 30 min at 37°C. When added to blood samples, PILV reduced the bacterial load to 75% (Figure 6A), indicating an anti-infective role of PILV in human blood. Treatment of human blood with a NO inhibitor abolished OPK of Newman in the absence or presence of PILV (Figure 6A). Similar results were found when measuring the OPK of *S. aureus* in mouse blood (Figure 6B). Furthermore, the specific phagocytosis of *S. aureus* Newman was determined in macrophage cell line RAW264.7 and differentiated U937 cells. As anticipated, the NO inhibitor or cytochalasin D completely depleted the PILV-enhanced phagocytosis of *S. aureus* in either human or mouse macrophages (Figures 6C,D). Altogether, these data demonstrate that PILV promotes the phagocytic killing of *S. aureus* in a NO-dependent manner via arginase inhibition.

## Discussion

NO is a versatile effector that plays a central role in both, antimicrobial activity and immunomodulation. During an infection, the highly cytotoxic NO levels produced by innate immune cells limit pathogen growth (27), and although *S. aureus* has several genes for efficient NO detoxification, NO production is still critical in host resistance to the staphylococcal disease (28, 29). NO targets the Agr quorum sensing system to disrupt cell-to-cell communication in *S. aureus* (30) and acts as a signaling messenger to promote immune cell activity (27, 31). Inhibition of NOS in macrophages or neutrophils significantly blocks phagocytosis and intracellular killing, and increases survival of *S. aureus* (18, 32–34). These observations resulted in the development of NO delivery systems that harness the antimicrobial properties of this short-lived gas (27, 35). In the present study, we found that the administration of a combination of four metabolites during *S. aureus* infection enhanced endogenous NO production.

Metabolomics is an advanced technology that examines metabolic processes, identifying biomarkers and unraveling mechanisms (36). The analysis of crucial metabolites in samples with different statuses has virtually become a significant part of improving the diagnosis, prognosis, and therapy of diseases (37). Various metabolites, including glucose, malate, N-acetylglucosamine, myo-inositol, linoleic acid, L-proline, L-valine, and L-leucine, have shown extended protection against bacterial infections (14–18, 38). However, the mechanisms underlying their anti-infection properties are currently minimally understood. Studies showed an inhibitory effect of L-proline, L-isoleucine, L-leucine, and L-valine on arginase activities; this inhibition is relatively specific as other amino acids, such as glycine and L-glutamine, do not influence arginase activities (39–41). Arginase inhibition by L-valine increases NO production in endothelial cells and macrophages during lipopolysaccharide (LPS) treatment (18, 42). Arginase elimination in macrophages favors host survival in *Toxoplasma gondii* infections and reduces bacterial loads in lung infections with *Mycobacterium tuberculosis* (43). NOS and arginase are co-induced during staphylococcal infection but the enzymatic role of arginase is distinctly more robust than that of NOS. The induced arginase competes with NOS for L-arginine utilization, which limits the NO-mediated anti-infection effect (26). Interestingly, L-proline, L-isoleucine, L-leucine, and L-valine boosted NO production via arginase inhibition in *S. aureus*-infected hosts. More importantly, the combined administration of all four metabolites provided stronger arginase inhibition and protection against *S. aureus* infection. This happened largely through a mechanism where in the absence of arginase activity, L-arginine is mostly consumed by NOS to produce NO.

There are two isoforms of arginase, arginase I and arginase II, which are located on cytosol and mitochondria, respectively. Branched-chain amino acids (BCAAs) cause significant inhibition of arginase I and only a minor effect on arginase II, while L-proline displays a stronger inhibition of arginase II in comparison to arginase I (39). This possibly explains the stronger arginase inhibition caused by the combination of L-proline and BCAAs but not by a 4-fold concentration of each metabolite, although further investigation is still required on this matter. Additionally, *S. aureus* carries its own arginase gene, which might behave like its host counterpart reducing the amount of L-arginine available for NOS (12, 44, 45). The inhibitory effect of L-proline and BCAAs on staphylococcal arginase needs to be determined in the future.

The fact that the host employs L-proline and BCAAs as anti-infection metabolites against *S. aureus* bloodstream infection is surprising because *S. aureus* presents amino acid auxotrophies in minimal media regarding L-proline, L-valine, and L-leucine, although its genome contains the gene sets for the biosynthesis of these amino acids (46, 47). However, our results showed an elevation in these amino acids in the plasma of infected animals. Instead of enhancing *S. aureus* growth, exogenous supplementation of L-proline and BCAAs facilitated the phagocyte-mediated OPK of *S. aureus* and the elimination of staphylococci by the host. Consistently, *S. aureus* infection reduces the transcriptional level of BCAA transaminase 2, which mainly contributes to BCAA degradation (48), suggesting a possible lower degradation rate of BCAAs in infected mice. TLR2/TLR6 agonists stimulate a significant increase in L-valine and L-isoleucine in mice (8), revealing that *S. aureus*-derived lipoteichoic acid and peptidoglycan could be the inducer in infected animals. The qRT-PCR data showed that *S. aureus* infection has no impact on TLR2/TLR6 expressions but exogenous L-isoleucine, L-valine, or PILV significantly reduces the level of TLR2 in blood samples (Figure S5). The down-regulated expression of TLR2 would cause a weaker immune response from lipoteichoic acid and peptidoglycan stimulations. Thus, in the presence of exogenous L-isoleucine, L-valine, or PILV, the level of proline and BCAAs may not be enhanced by *S. aureus* infection if the TLR2/TLR6-dependent mechanism has been demonstrated. This also seems logical since exogenous addition has been able to meet the demand for boosting NO to kill *S. aureus*, and those biomarkers no longer need to be increased through TLR2/TLR6-dependent mechanism. The reduction of TLR2 expression by L-isoleucine, L-valine, or PILV may have another effect. The inflammation is a double-edged sword; too little is not enough to control infection, and too high would have damage to the body. Thus we presume that the exogenous L-isoleucine, L-valine, or PILV inhibits the inflammation in a TLR2 dependent manner while still boosting NO to kill *S. aureus*. The specific mechanism for this phenomenon remains to be determined in future studies for a better understanding of the metabolite-mediated innate immunity against *S. aureus* infection. Besides the innate immunity, the lymphocyte response-related adaptive immunity also can be potentially regulated by L-proline and BCAAs to enhance its anti-infectivity. BCAA not only increases fuel sources for CD4^+^ and CD8^+^ T cells and the secretion of intestinal immunoglobulins, but also regulates the development of Treg (regulatory T) cells through mTORC1 signaling (49, 50). The proliferation of activated T cell is rely on the uptake of L-proline (51). The role of BCAA and L-proline on adaptive immunity *against S. aureus* infection should be evaluated in future studies.

## Data Availability Statement

All datasets generated for in this study are included in the article/Supplementary Material.

## Ethics Statement

The studies involving human participants were reviewed and approved by Institutional Ethics Committee at the Guangdong Institute of Microbiology. The patients/participants provided their written informed consent to participate in this study. The animal study was reviewed and approved by Institutional Animal Care and Use Committee at the Guangdong Institute of Microbiology (Animal Welfare Assurance Number GT-IACUC201907031).

## Author Contributions

XC, RP, and YS developed methods and conceptualized ideas. RP, YS, HZ, and XC designed experiments. XC, RP, YS, and YH performed experiments. XC and RP analyzed data. XC, YS, and RP wrote the manuscript. All authors contributed to the article and approved the submitted version.

## Conflict of Interest

The authors declare that the research was conducted in the absence of any commercial or financial relationships that could be construed as a potential conflict of interest.
