# Supplementary material for: Inhibition of Host Arginase Activity Against Staphylococcal Bloodstream Infection by Different Metabolites
Source: Front Immunol. 2020 Jul 28;11:1639. doi: 10.3389/fimmu.2020.01639 (PMC7399636; doi:10.3389/fimmu.2020.01639)
Supplement: Supplementary file 1 [file Data_Sheet_1.docx]

**Figure S1.** **An increasing dose of L-proline is not able to increase survival.**


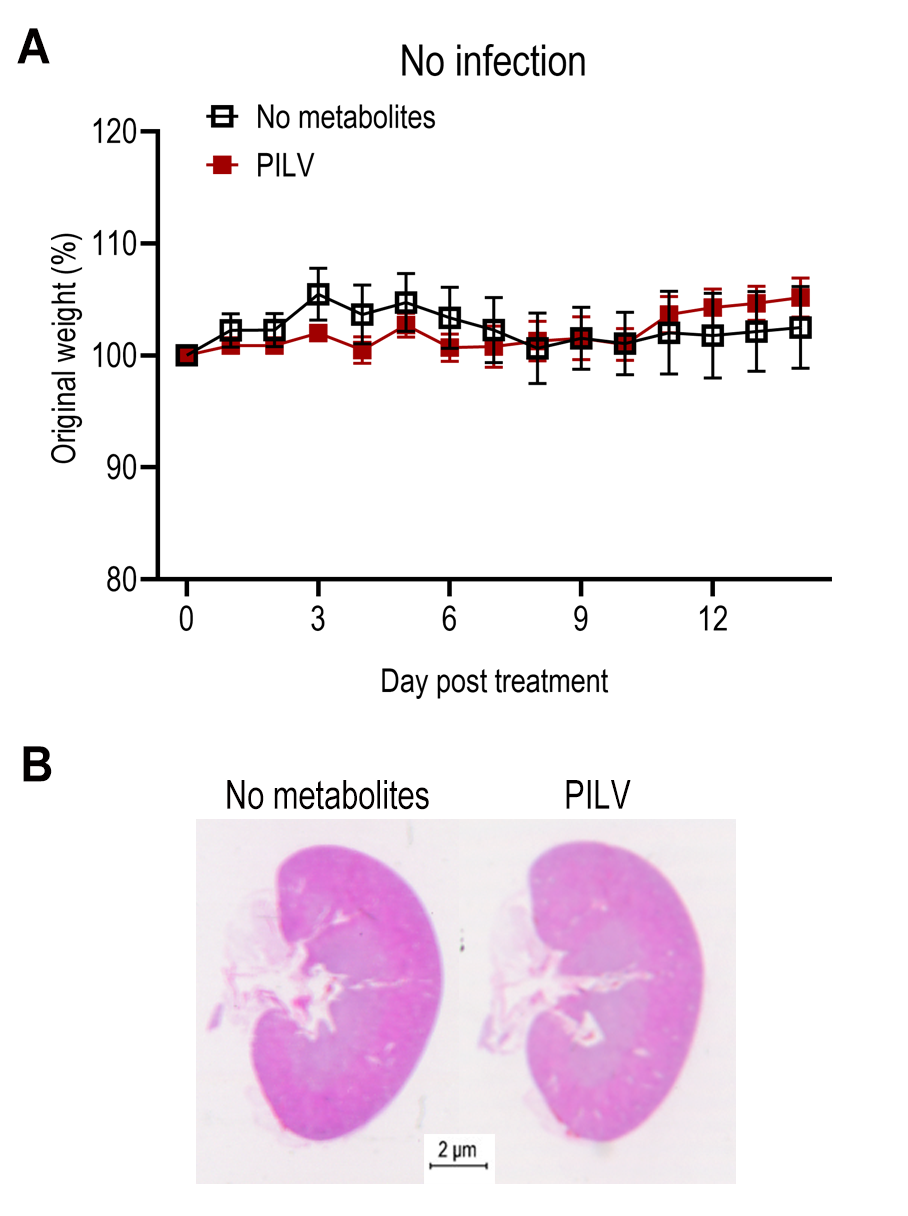


**Figure S2. The PILV combination treatment had no influence on the body weight (A) and kidney phenotype of mice (B) without *S. aureus* infection.**

**Figure S3. Metabolite treatments are not able to increase NO release in the mouse without *S. aureus* infection.**


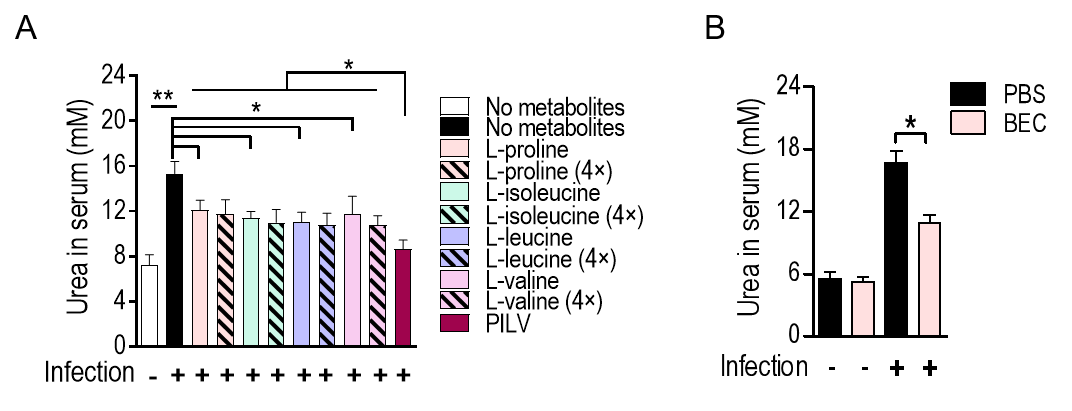


**Figure S4. Measurement urea production in mouse serum injected with *S. aureus* USA300**. **(A)** Staphylococcal infection-induced urea content was reduced by single metabolite treatment and further decreased by PILV treatment. **(B)** A competitive arginase inhibitor, BEC (S-(2-boronoethyl)-L-cysteine), was able to inhibit urea production Data are represented as Error bars ± SEM. * *P* < 0.05 and ** *P* < 0.01.


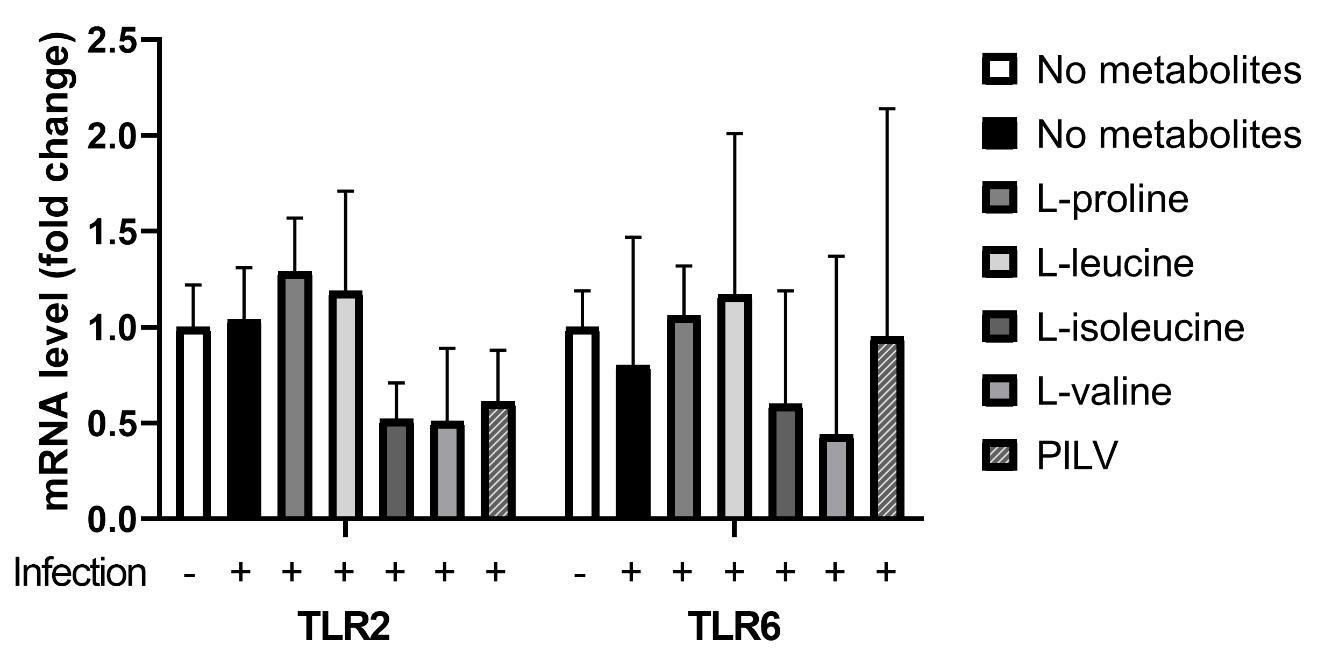


**Figure S5. The mRNA levels of TLR2 and TLR6 in mouse blood samples.**

**Table S1 The primers used in this study**

| Primer name | Sequence | Tm (℃) | Product length | Purpose |
| --- | --- | --- | --- | --- |
| *Arg1*-F | TGAGCTTTGATGTCGACGGG | 58 | 237 | qRT-PCR test for gene *Arg1* |
| *Arg1*-R | GTTGAGTTCCGAAGCAAGCC | 58 |  |  |
| *Arg2*-F | TCGGGGACAGAAGAAGCTAGGA | 58 | 204 | qRT-PCR test for gene *Arg2* |
| *Arg2*-R | ACTTCAGCCAGTTCCTGGTTGG | 61 |  |  |
| *Nos1*-F | CTGGAGACCACCTTCACAGG | 58 | 167 | qRT-PCR test for gene *Nos1* |
| *Nos1*-R | GCATGCTGAGGTCCGTTACT | 59 |  |  |
| *Nos2*-F | CAGTCCTCTTTGCTACTGAGACAGG | 62 | 201 | qRT-PCR test for gene *Nos2* |
| *Nos2*-R | TCTTCAGAGTCTGCCCATTGCT | 61 |  |  |
| *Nos3*-F | CCTTCACCCACTGAGCAGCTATT | 61 | 233 | qRT-PCR test for gene *Nos3* |
| *Nos3*-R | TGCAGCTTTCCCCACTGGAT | 60 |  |  |
| *Actb*-F | CAAGAGAGGTATCCTGACCCT | 58 | 188 | qRT-PCR test for gene *Actb* |
| *Actb*-R | TGATCTGGGTCATCTTTTCAC | 56 |  |  |
| *Tlr2*-F | GCTCTGGCTCAAATCCTGGT | 60 | 257 | qRT-PCR test for gene *Tlr2* |
| *Tlr2*-R | GGAGCTTTCTTGGGCTTCCT | 59 |  |  |
| *Tlr6*-F | CTGAGCTTCGGATGCCTGAT | 60 | 262 | qRT-PCR test for gene *Tlr6* |
| *Tlr6-R* | CCCAGGAAAGTCAGCTTCGT | 60 |  |  |
